# Effect of Lignin Incorporation on Properties of Polyester Films of Lignin-Grafted-PCL/PLGA/PLA Polymers as Packaging Materials

**DOI:** 10.1021/acsomega.5c13155

**Published:** 2026-05-28

**Authors:** Omar Mendez, Carlos Astete, Rafael Cueto, Fannyuy Kewir, Jessica Eberhard, Thanida Chuacharoen, Olivia Springer, Marie Howe, Cristina Sabliov

**Affiliations:** a Biological & Agricultural Engineering, Louisiana State University and LSU Ag Center, Baton Rouge 70803, United States; b Chemistry, 5779 Louisiana State University, Baton Rouge 70803, United States; c Biological Sciences, 5779 Louisiana State University, Baton Rouge 70803, United States; d Faculty of Science and Technology, 65111 Suan Sunandha Rajabhat University, Bangkok 10300, Thailand

## Abstract

This article reports on the effect of lignin on the properties of films made from lignin (LN) grafted to either PCL, PLGA, or PLA. LN-PCL/PLGA/PLA polymers were synthesized by an acylation reaction, and lignin grafting was confirmed using FTIR and H NMR spectroscopy. Films were made from the grafted polymers with a solvent-casting technique. The thermal, mechanical, and functional properties of the films were measured using standard methods and compared against the properties of the polyester films without lignin. Thermal analysis of the films showed glass transition temperatures of 55.9, 46.2, and 56 °C for LN-PCL, LN-PLGA, and LN-PLA, similar to those of the free polyesters. LN grafting reduced strain at break and yield strength, particularly when grafted to PLA, while having minimal impacts on PCL and PLGA. All LN-PCL/PLGA/PLA films presented an UV transmittance below 15%, an improvement over those of the polyesters measuring 60% and up, showing a high potential as a UV-shielding material. Contact angle analysis indicated no effect on the wettability of the films from incorporating lignin, with an average water contact angle of 82.71 ± 8.42°. While permeability was similar across polymers, ranging from 3.6 × 10^12^ to 8.5 × 10^–12^ g/Pa*s*m for PLGA and PCL films, that of LN-PLA was significantly higher (1.5 × 10^11^ ± 1.9 × 10^–12^g/Pa*s*m), but in the same range. In summary, the lignin films had comparable properties to those of the neat polyesters while having higher UV-shielding properties, indicating the potential of lignin-grafted materials as biodegradable alternatives for packaging UV-sensitive products.

## Graphical abstract

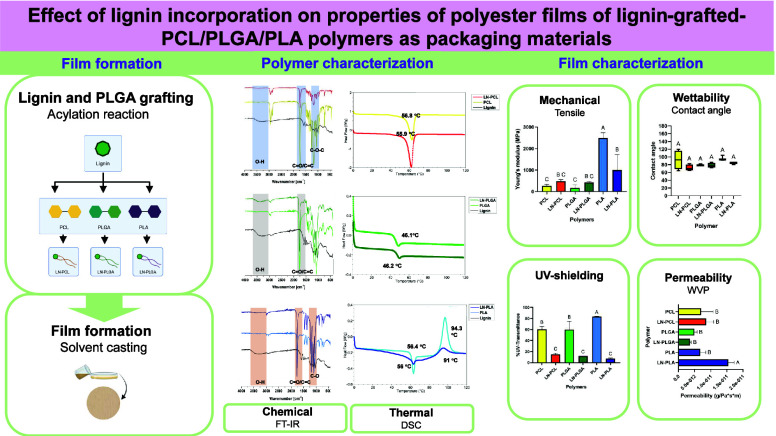

## Introduction

Of the 140 million tons of plastic produced annually, 40% is produced by the packaging industry. The widespread use of single-use petroleum-derived plastics as food packaging has contributed significantly to environmental pollution.^1^ The significant environmental impact of nonbiodegradable, petroleum-based polymers has incentivized a shift toward sustainable alternatives. Biodegradable, natural, or synthetic polymers are promising candidates to replace conventional plastics. Natural polymers, derived from renewable resources or waste byproducts, can be composted and are nontoxic and biocompatible.^2,3^

Lignin is the most abundant, natural, and aromatic biopolymer, with a significant potential for value-added applications due to its intrinsic UV-absorbing, antioxidant, and antibacterial properties.^1,4^ Lignin is produced in vast quantities as a byproduct of the paper and bioethanol industries. Nevertheless, lignin, in its majority, is used as low-value fuel.^5^

Some efforts have been made to develop lignin composites with other polymers by mixing lignin to improve the oxidative stability, UV-barrier properties, and mechanical properties of newly generated materials.^6–12^ Lignin addition to other natural polymers, such as starches and chitosan, was done with the goal of forming biodegradable and cost-effective materials with improved mechanical strength, resistance, and barrier properties.^13–15^ Alternatively, lignin was grafted with polyesters, such as polycaprolactone (PCL), via ring-opening polymerization or acylation reaction for the fabrication of composite films with high UV absorption.^6,16,17^ Lignin-graft-PLGA nanoparticles have been used in veterinary medicine as a delivery system for florfenicol, enhancing the antimicrobial activity at reduced concentrations and decreasing toxicity of the antibiotic.^18^ PLA has also been covalently grafted into lignin via ring-opening polymerization, resulting in nano PLA-lignin particles with improved dispersion in PLA matrixes.^19^ LN-*g*-PLA polymers have exhibited increased tensile strength,^20^ while physical blends have decreased tensile strength when mixed with lignin.^21^

Polyesters, such as polycaprolactone (PCL), poly­(lactic-*co*-glycolic) acid (PLGA), and poly lactic acid (PLA), are suitable for lignin grafting and food packaging applications because of their balance between biodegradability and mechanical performance.^22–24^ PCL is an aliphatic polyester, which possesses a slow degradation rate and has a high biocompatibility. PCL has been widely used in biomedical applications, such as drug delivery, tissue engineering, and biodegradable implants.^3^ In the food industry, the manufacture of porous or dense films from PCL has been proposed recently for protection of bioactive components.^22^ PLGA is a copolymer composed of lactic and glycolic acid monomers. It is a versatile, biodegradable, and biocompatible polymer with tunable chemical and physical properties that can be adjusted by changing parameters like the molecular weight and lactic acid vents:glycolic acid ratio. It can be dissolved in multiple solvents including acetone, tetrahydrofuran, and chlorinated solvents, and is easily degraded by hydrolysis.^25^ PLA has mechanical properties comparable to those of petroleum-based polymers, and it is the most widely used biopolymer in the food industry because of its production through fermentation of crops like sugar cane, maize, and potato. It is classified as a generally recognized as safe (GRAS) material with high biocompatibility.^24^

The purpose of this study is to form and evaluate the physicochemical and functional properties of films synthesized from lignin grafted with PCL, PLGA, and PLA, with the goal of assessing their potential for packaging applications, relative to their polyester counterparts. We hypothesized that lignin provided increased UV-barrier performance and improved water permeability while maintaining mechanical and thermal stabilities of polyester films. To confirm our hypothesis, we synthesized LN-*g*-PCL/PGA/PLA polymers, and after FTIR and NMR characterization, the polymers were used to produce films by a solvent-casting process. The thermal, mechanical, and functional properties of the films with and without lignin were measured using standard methods.

## Experimental Section

### Materials

Alkaline lignin (ALN) was purchased from TCI (Portland, OR). Oxalyl chloride, dimethylformamide (DMF), dimethyl sulfoxide (DMSO), and potassium nitrate were obtained from Fisher Scientific (Fair Lawn, NJ). Toluene was obtained from Mallinckrodt (Hazelwood, MO). Dichloromethane (DCM) was purchased from Supelco (Burlington, MA), and poly­(lactic-*co*-glycolic) acid (PLGA) (1:1) (35,000–45,000 Da) was acquired from Sigma-Aldrich (St. Louis, MO). PLA (45,000 to 55,000 DA) was procured from PolySciTech (Akina Inc., West Lafayette, IN). Polycaprolactone (PCL) (50,000 DA) was purchased from Polysciences Inc. (Warrington, PA). Deionized (DI) water was obtained from a Barnstead Smart2Pure water purification system (Barnstead International, Dubuque, IA). Silicate was from Fisher Scientific (Fair Lawn, NJ).

### Polymer Synthesis

LN-*g*-PCL/PLGA/PLA polymers were synthesized with an acylation reaction, following a protocol available in the literature^16^ adapted for the polyesters of choice (Figure 1). Four grams of either PCL, PLGA, or PLA were dissolved in 75 mL of dichloromethane (DCM) and mixed until completely dissolved. 110 μL of oxalyl chloride and 4 mL of dimethylformamide were added to the solution and mixed for 5 h. The solution was concentrated using a R-300 Rotavapor (Buchi Corporation, New Castle, DE) and redissolved into 30 mL of dimethyl sulfoxide (DMSO), except for PLA, which was left dissolved in DCM. In a separate flask, 1 g of lignin was dissolved in 30 mL of DMSO. Both solutions were combined and mixed for 24 h. The lignin-grafted polymers were precipitated in 30 mL of ethyl ether and washed repeatedly with ethyl ether. Methanol was used for washing and removal of unreacted lignin. The polymer was frozen at −80 °C and then freeze-dried in a Labconco FreeZone 2.5 Plus (Labconco, Kansas City, MO).

**Figure 1:**
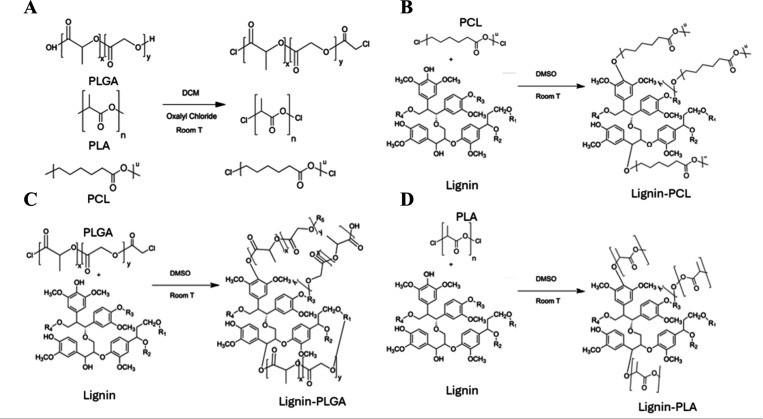
Lignin grafting reaction scheme: (A) first-step acylation on polyesters, (B) lignin-PCL grafting, (C) lignin-PLGA grafting, and (D) lignin-PLA grafting.

### Film Formation

Films were cast using a solvent evaporation method with solvents suitable for each grafted polymer. The LN-*g*-PCL/PLGA/PLA polymer (400 mg) was dissolved in 20 mL of DCM for PLA and PLGA or 30 mL of toluene for PCL. The solution was cast on 120 mL of PTFE Petri dishes and left evaporating for 24 h under a fume hood.

### Fourier Transform Infrared (FTIR)

FTIR spectroscopy was used to confirm the grafting of lignin to the PCL/PLGA/PLA polymers. Dried polymer transmittance was analyzed at a resolution of 4 cm^–1^ between 4000 and 600 cm^–1^ using a Bruker Tensor 27 instrument (Bruker 500, Billerica, MA).

### Hydrogen Nuclear Magnetic Resonance (H NMR)

H NMR was used to detect the chemical changes and confirm the grafting of the lignin to the PCL/PLGA/PLA polymers. Samples of the polymers were dissolved into DMSO at a concentration of 10 mg/mL. H NMR analyses were performed using a Bruker 400 NMR spectrometer (Bruker, Billerica, MA).

### Thermogravimetric Analysis (TGA)

Polymer composition was analyzed with TGA. Measurements were conducted on a TA Discovery TGA550 (New Castle, DE) with a heating rate of 50 °C/min, and a final temperature of 600 °C. Nitrogen gas was used as an oxidation agent. Data were collected using a TRIOS software program (TA Instruments, New Castle, DE) and analyzed with the Universal Analysis 2000 program (TA Instruments, New Castle, DE). The composition of the films was determined by the decomposition of the polymer into three components: water, bulk, and ash. Lignin % was calculated with the following equations.

$$\mathrm{ash}\%=\frac{\mathrm{ash}\;\mathrm{fraction}}{\mathrm{ash}\;\mathrm{fraction}+\mathrm{bulk}\;\mathrm{fraction}}\times 100$$

$$\mathrm{LN}\%=\frac{\mathrm{ash}\%\;\mathrm{of}\;\mathrm{film}}{\mathrm{ash}\%\;\mathrm{of}\;\mathrm{LN}}\times 100$$

### Differential Scanning Calorimetry (DSC)

Glass transition temperature was determined by DSC analysis using a Discovery DSC 250 (TA Instruments, New Castle, Delaware). Samples were prepared by cutting films into samples amounting to 5–10 mg. Films were placed inside aluminum pans and heated within a range of 0–120 °C at a rate of 10 °C/min. DSC was performed with an initial heating cycle, a cooling cycle, and a second heating cycle using the same heating range and rate as the first heating cycle. Results were collected and analyzed using the Universal Analysis 2000 program (TA Instruments, New Castle, DE).

### Mechanical Characterization

A tensile mechanical test was performed following the ASTM D882 test method for thin plastic sheeting with modifications. Films were cut into specimens with a 30 × 10 mm strip for testing. Thickness was measured using a 0–1″ micrometer (Anytime Tools, Granada Hills, CA). The tensile test was performed on a universal testing system, series 5969 (INSTRON, Norwood, MA), with a rate of separation of 12.5 mm/min. Strain at break point, yield strength, yield strain, and Young’s modulus were calculated from strain/stress curves.

### UV Transmittance

The absorption of ultraviolet (UV) light was measured with a transmittance test. Pieces of 20 × 20 mm were cut from the films; the thickness of the pieces was measured with a 0–1″ micrometer (Anytime Tools, Granada Hills, CA). A UV lamp Intelli-ray 400 (Uvitron Internacional Inc., West Springfield, MA) was used as the source of UV light. UV transmittance was measured using a UV–C (100–280 nm) digital meter (General Tools, Secaucus, NJ). UV transmittance was calculated with the following equation:

$$\mathrm{UV}\;\mathrm{transmittance}\;\%=\frac{\mathrm{UV}\;\mathrm{with}\;\mathrm{film}}{\mathrm{UV}\;w/o\;\mathrm{film}}$$

where UV w/o is the UV light emitted by the lamp detected without any obstruction. UV with film is the UV light detected with a film between the lamp and sensor.

### Wettability

Wettability of the films was determined by their contact angle (CA). 15 × 15 mm pieces were cut from the film, and the contact angle was measured using a sessile drop test. Drops of 15 μL volume were poured on top of the films and measured for 10 s using an Attention Theta optical tensiometer (Biolin Scientific, Beijing, China).

### Water Vapor Permeability

Water vapor permeability of the films was measured following the standard method for materials, ASTM E-96.^26^ Films were cut into circular pieces of 3.6 cm, and thickness was measured using a 0–1″ micrometer (Anytime Tools, Granada Hills, CA). Cups were filled with 100 g of dry silicate and assembled with the films in triplicates along with a triplicate of control cups without the films. The samples were placed inside a desiccator with a saturated KNO_3_ to maintain a constant relative humidity of around 80%. Samples were weighed periodically over a period of 10 days.

The water vapor transmission rate and permeance were calculated with the following equation:

WVT=G/tA

where *G* is the weight change from a straight-line section of the change-in-weight time graph (g). *t* is the time (h). *A* is the test area (cup mouth area) (m^2^). WVT is the water transmission rate (g/h*m^2^).

$$\mathrm{permeance}=\mathrm{WVT}/S(R_{1}-R_{2})$$

where *S* is the saturation vapor pressure in mm Hg (1.333 × 10^2^ Pa), from water vapor saturation tables. *R*_1_ is the relative humidity expressed as a fraction of the test chamber. *R*_2_ is the relative humidity of the vapor sink.

### Statistical Analysis

Mechanical analysis results were analyzed with Tukey tests and 2-way Anova. UV transmittance, wettability, and permeability were analyzed following a Tukey test, and multiple *t*-test analysis was performed on the permeability results. GraphPad Prism 10.5.0 (San Diego, CA, USA) software was used for all statistical analyses considering a significant effect when *P* > 0.05.

## Results and Discussion

### Chemical Characterization

The LN-PCL/PLGA/PLA polymers were synthesized following a two-step acylation reaction by activation with oxalyl chloride in the first step and linking lignin to the polymer in the second step. FTIR and NMR analyses were used to corroborate the bonding of lignin to the polymers.

Lignin-specific peaks, corresponding to α-carbonyl aromatic ring 1520–1540 and 1530–1580 cm^–1^ vibrations, were found in the neat lignin and retained in LN-PCL/PLGA/PLA polymers (Figure 2). Similar FTIR spectra were found by ref 16 where PLGA was grafted onto alkaline and lignosulfonate lignin. As previously reported, we also found a peak indicative of carbonyl groups (C=O) between wavenumbers 1700 to 1800 cm^–1^ (Figure 2B,C), in both PLGA and PLA spectra. The same peak was found in the LN-PLGA/PLA polymers corroborating the presence of esterified carbonyl groups and, consequently, PLGA, and PLA in their respective LN-grafted polymers. This ester C=O band shows a pronounced peak in the LN-PCL spectrum (Figure 2A), and together with the shift in the broad band (3000–3700 cm^–1^) attributed to O–H vibrations, confirms successful covalent grafting of PCL onto lignin. Additionally, the characteristic C–O stretching at 1000–1250 cm^–1^ in PLA could be identified in the LN-PLA spectrum (Figure 2C).

**Figure 2:**
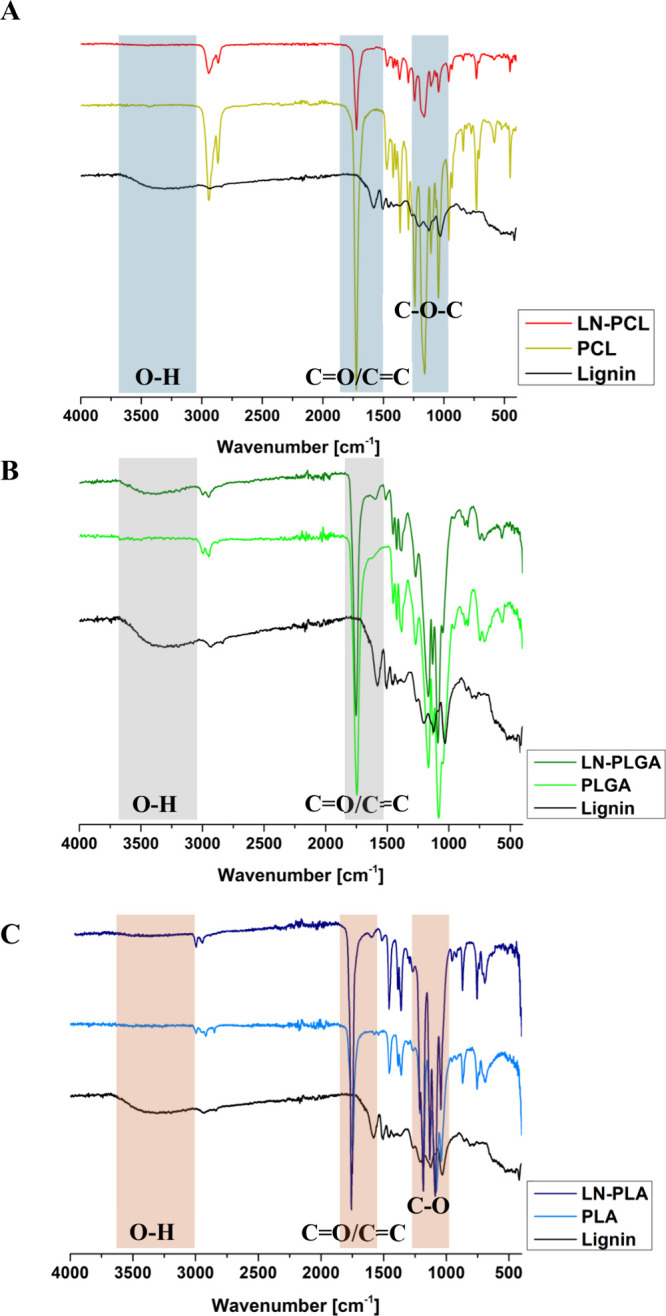
FTIR analysis for panel (A). LN-PCL polymer, relative to LN and PCL; (B) LN-PLGA polymer, relative to LN and PLGA; and (C) LN-PLA polymer, relative to LN and PLA.

^1^H NMR plots for LN-PCL/PLGA/PLA polymers show aromatic hydrogens within a range of 6.5–7.7 ppm in lignin and all the lignin-grafted polymers (Figure 3). The characteristic peaks of water and DMSO at 3.3–3.4 and 2.5 ppm respectively were identified in all samples. Peaks corresponding to lactide groups CH and CH_2_ were found at 5.1–5.2 and 4.6–4.8 ppm in both LN-PLGA and LN-PLA polymers (Figure 3B,C). Methoxy groups −OCH_3_ were observed at 3.5–4.0 ppm for lignin and all the lignin-grafted polymers (Figure 3A–C). Also, lactide methyl groups present in both PLGA and PLA polymers and their corresponding grafted polymers were present at around 1.5–1.7 ppm (Figure 3B,C). Other studies have shown alike NMR spectra for lignin-grafted polymers: LN-PCL,^6^ LN-PLGA,^16^ and LN-PLA.^27^ Hence, ^1^H NMR, in addition to FTIR data, indicates a successful grafting of lignin to the neat polyesters.

**Figure 3:**
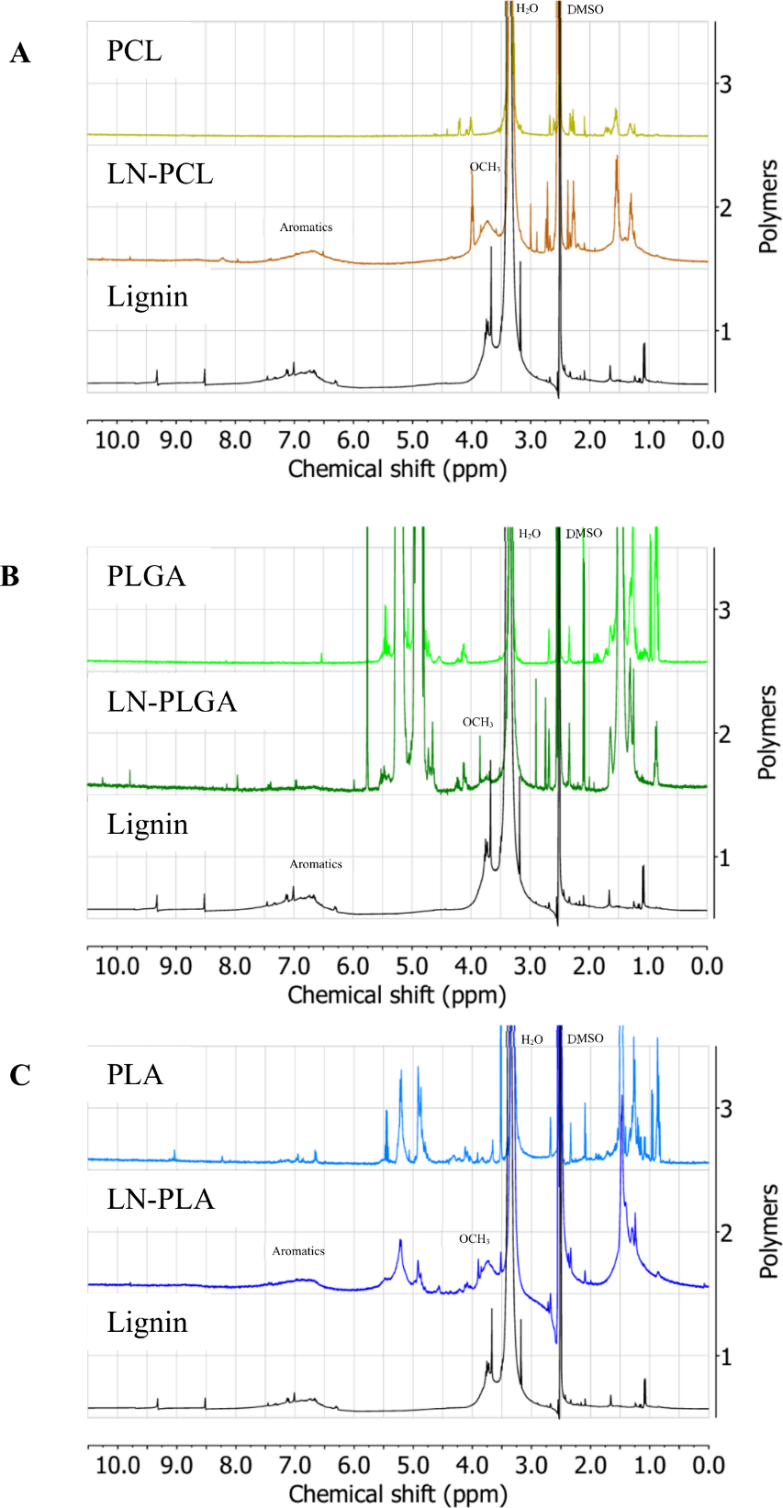
^1^H NMR analysis for lignin-PCL/PLGA/PLA polymers. (A) Lignin-PCL relative to lignin and PCL, (B) lignin-PLGA relative to lignin and PLGA, and (C) lignin-PLA relative to lignin and PLA.

### Thermogravimetric Analysis

The polymer synthesis process was performed with a lignin-to-PLA/PLGA/PCL (w/w) ratio of 1 to 4, and the final lignin content in the grafted polymer was estimated by thermogravimetric analysis (TGA) (Figure 4). Because lignin had a high residue content (73.1 ± 1.8%), and the neat polymers showed a low residue content (PLA 1.2 ± 0.1%, PLGA 1.7 ± 0.2%, and PCL 7.8 ± 0.8%), the concentration of residue was used to quantify the lignin % in the grafted polymers (Figure 5). Based on the initial residue content in lignin, the lignin % in the LN-PCL, LN-PLGA, and LN-PLA polymers were calculated as 3.3 ± 0.02, 13.2 ± 4.8, and 20.5 ± 0.7%, respectively. The solubility of the polyesters in the DMSO could affect the degree of grafting onto lignin during the reactions and hence the final amount of lignin in the LN-PCL/PLGA/PLA polymers. The glass transition temperature was determined by differential scanning calorimetry (DSC) analysis. PCL and LN-PCL polymers had similar glass transition temperatures (*T*_g_) (56.8 and 55.9 °C), indicating that grafting PCL to lignin did not have a significant effect on the *T*_g_ of the polymer (Figure 6A), as reported in other studies.^17,28^ Similarly, LN-PLGA polymer had a *T*_g_ of 46.2 °C, not much different from PLGA (46.1 °C) (Figure 6B), and PLA and LN-PLA films showed a similar *T*_g_ (56.4 and 56 °C, respectively) (Figure 6C).

**Figure 4:**
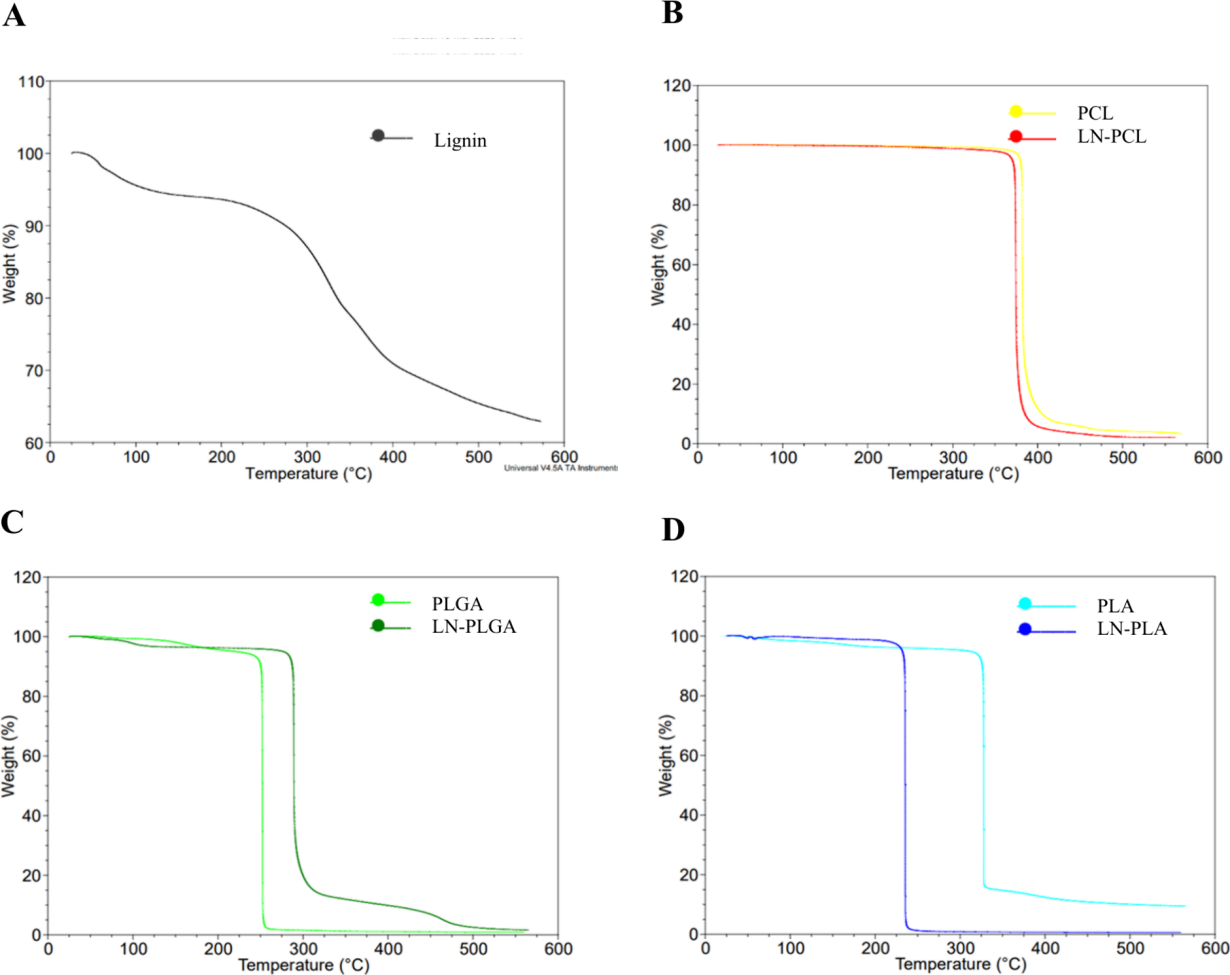
Thermographic decomposition analysis: (A) lignin, (B) PCL and LN-PCL, (C) PLGA and LN-PLGA, and (D) PLA and LN-PLA.

**Figure 5:**
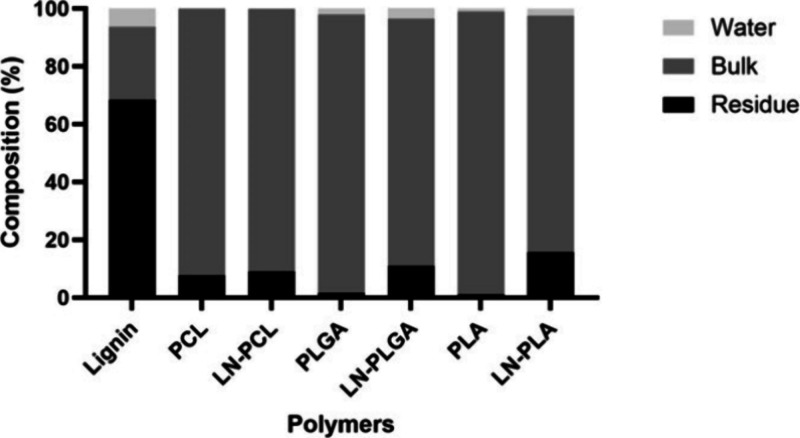
Thermogravimetric composition analysis for lignin-*g*-(PCL/PLGA/PLA) polymers (*n* = 3).

**Figure 6:**
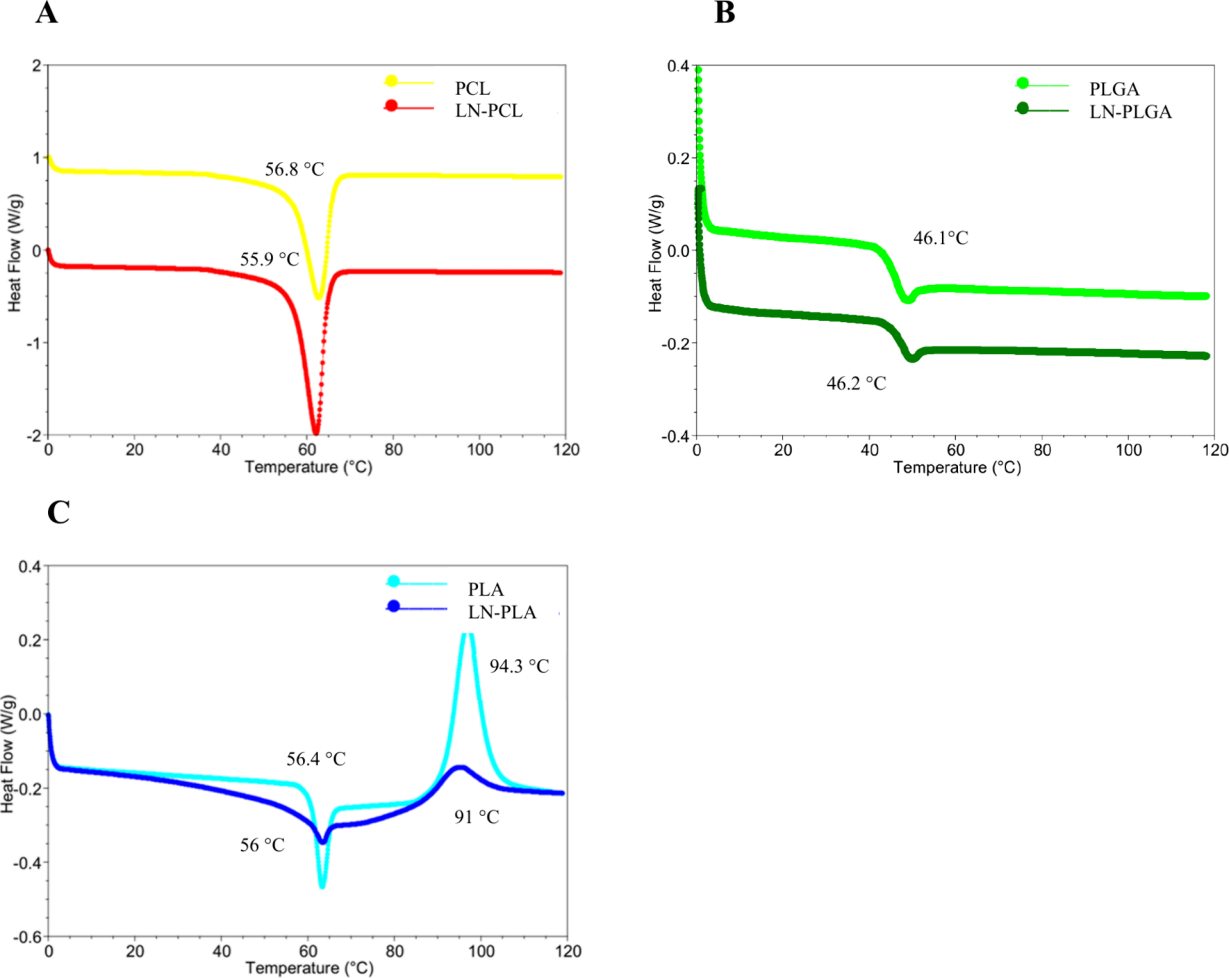
Differential scanning calorimetry (DSC) analysis of polymer films: (A) PCL and LN-PCL, (B) PLGA and LN-PLGA, and (C) PLA and LN-PLA.

Commercial PLA is usually rich in L-PLA, associated with a high level of chain order leading to crystallization under the correct thermal conditions.^29^ While both PLA and LN-PLA showed similar cold crystallization temperatures (*T*_cc_) (94.3 and 91 °C), LN-PLA films showed smaller exothermic peaks. This suggests that the presence of lignin may inhibit the crystallization of PLA, behaving as a toughening agent and preventing nucleation of the PLA chains.^30^

### Mechanical Characterization

All films had a thickness below 150 μm (Table 1). PCL and LN-PCL thickness were not significantly different. Thickness was also not significantly different between PLA and LN-PLA films. The only significant difference was found between the PLGA and LN-PLGA films. The mechanical properties of the films were measured following the ASTM D882.^31^ Strain at break point (mm/mm), yield strain (mm/mm), yield strength (MPa), and Young’s modulus (MPa) are depicted in Figure 7. Anova analysis showed that while both the presence of lignin and polymer type significantly impacted the mechanical properties of the films, the polymer type had a stronger impact than lignin addition (Table 2).

**Table 1:** Thickness (mm) of LN-(PCL/PLGA/PLA) Polymers, Tukey Test Differences Were Indicated by *P* < 0.05 (*n* = 3)

polymer	PCL	PLGA	PLA	LN-PCL	LN-PLGA	LN-PLA
thickness (μm)	76.8 ± 17.9^ac^	101.4 ± 10.2^a^	56.7 ± 19.2^bc^	87.3 ± 4.3^ab^	35 ± 2.4^c^	79.5 ± 24.9^ab^

**Table 2:** Two-Way Anova for the Mechanical Analysis of Lignin-Grafted Polymer Films: * (*P* < 0.05); ** (*P* < 0.005); *** (*P* < 0.0005) (*n* = 3)

parameter	strain at break	yield strain	yield strength	Young’s modulus
lignin	0.0218*	0.4713	0.015*	0.0054**
polymer	0.0049**	0.0011**	<0.0001***	<0.0001***
interaction	0.2102	0.3734	<0.0001***	<0.0001***

**Figure 7:**
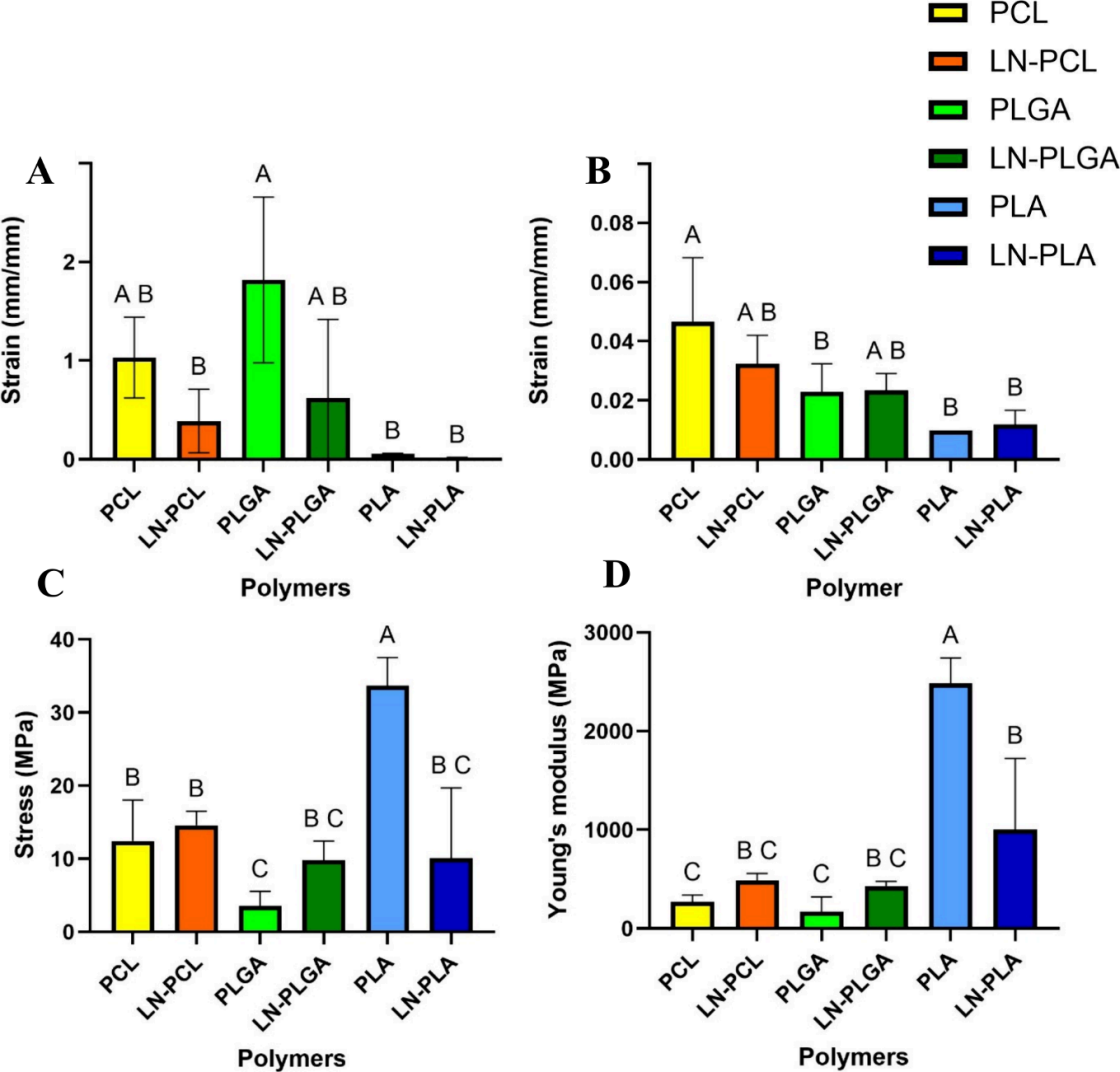
Mechanical characterization of LN-(PCL/PLGA/PLA) polymer films. (A) Strain at break point, (B) yield strain, (C) yield stress, and (D) Young’s modulus. Tukey test differences were indicated by *P* < 0.05, *n* = 3.

Small, yet significant differences were found across polymer types, with PLGA having the highest strain at break (1.82 ± 0.84 mm/mm), followed by PCL (1.03 ± 0.41 mm/mm), and PLA (0.17 ± 0.17 mm/mm). Overall, lignin-grafted polymers had a lower strain at break compared to the neat polyesters (Figure 7A). Yield strain was the highest for PCL and LN-PCL films (0.05 ± 0.02 and 0.03 ± 0.01 mm/mm), followed by PLGA and LN-PLGA films (0.02 ± 0.01 and 0.02 ± 0.00 mm/mm) and PLA and LN-PLA (0.02 ± 0.01 and 0.01+ 0.00 mm/mm), with all values in a similar range (Figure 7B).

LN addition to PCL and PLGA did not significantly impact yield strength (12.44 ± 5.62 versus 14.58 ± 1.95 MPa for PCL and LN-PCL, respectively, 3.59 ± 1.95 MPa versus 9.82 ± 2.6 MPa for PLGA and LN-PLGA, respectively). However, the grafting onto lignin decreased the PLA yield strength from 23.52 ± 14.88 to 10.07 ± 9.64 MPa (Figure 7C). Young’s modulus followed the same trend (Figure 7D).

As reported in the literature, films made from PCL grafted with lignin exhibited similar mechanical properties to PCL regardless of the presence of lignin;^32^ grafting of lignin to PCL showed an increased tensile strength exclusively at higher molecular weight (*M*_w_) due to a greater number of covalent bonds formed. This correlates with our results showing nonsignificant differences in strain and Young’s modulus between the PCL and LN-PCL films. Similarly, no significant differences were observed between PLGA and LN-PLGA films and these results also are corroborated by one of our previous studies^33^ in which PLGA was grafted onto two different types of lignin (alkaline lignin and sodium lignosulfonate), and (A/S)­LN-PLGA did not show significant differences when grafted to PLGA at 1:4 w/w ratio. Strain at break and yield strain were not significantly different between PLA and LN-PLA films. However, PLA films demonstrated both a higher yield strength and Young’s modulus. Similar results have been observed before^27^ in which an addition of 10% w/w of lignin nanoparticles to PLA films reduced both yield strength from around 30 to 25 MPa and Young’s modulus from 3000 to around 2000 MPa. Lignin has a high carbon content as well as low density and hardness, and because of this, it is usually considered as an additive to improve mechanical properties of polymers.^7^ Nevertheless, at increased amounts (>20%), it can lead to aggregation and weakening of the tensile properties.^7,34^ This correlates with the DSC findings as lignin can affect the crystallization process of PLA by interrupting the nucleation of the polymer chains^29^ thus decreasing the yield strength and Young’s modulus.

### UV Transmittance

UV shielding is an important factor in packaging applications. Photo-oxidation can be a source of chemical reactions leading to loss of nutritional value, product degradation, and mechanical deterioration of packaging materials.^35^ To protect materials from solar radiation, different synthetic organic and inorganic materials with UV-blocking properties have been developed and incorporated into packaging films. Specifically, the development of biodegradable UV filters is relevant to the development of biocompatible and green products.^36^

Lignin is considered one of the most significant, natural, UV-blocking materials.^35^ Lignin contains multiple chromophore functional groups that can absorb UV light in a range of 250–300 nm.^37^ Lignin has been added to multiple composites to increase their UV resistance, such as PVA films,^38^ lignin-graft-poly­(lauryl methacrylate),^39^ and in our previous work, we determined that its UV-shielding properties are able to remain constant for over 12 months in storage under various humidity conditions.^40^

The UV-blocking properties of the films were assessed with a transmittance test. Lignin showed a very significant reduction in the UV transmittance of the films. PCL films had a transmittance of 61 ± 5%; meanwhile, LN-PCL films measured 15 ± 2%. Similarly, PLGA (60 ± 15%) had a higher transmittance than LN-PLGA films (12 ± 0%). PLA films showed the highest transmittance (83 ± 1%), and LN-PLA showed the lowest (7 ± 1%). No significant differences were observed among all lignin-grafted polymers, but neat polymers showed differences in transmittance with PLA having the highest (Figure 8).

**Figure 8:**
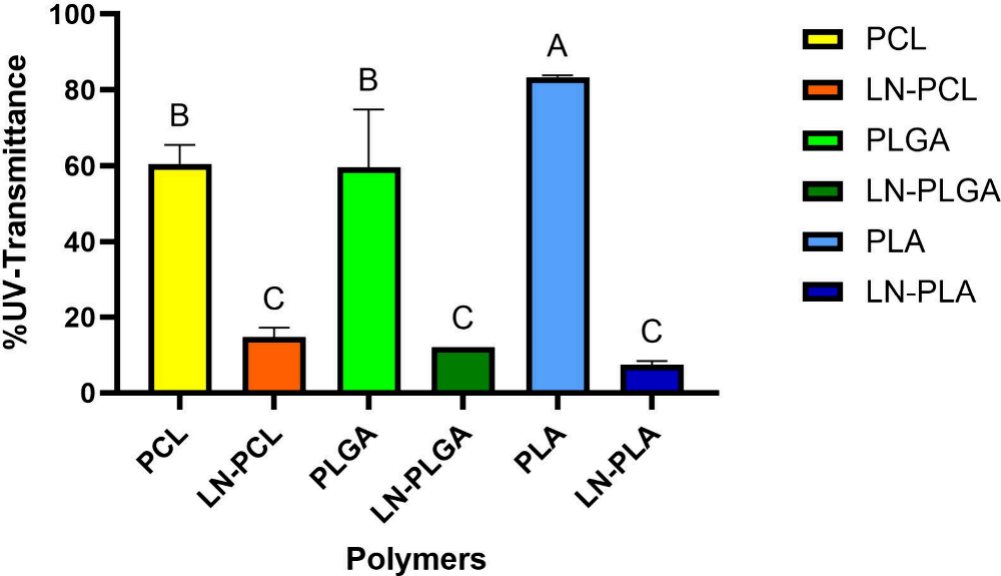
UV transmittance of LN-(PCL/PLGA/PLA) polymer films. Tukey test differences were indicated by *P* < 0.05, *n* = 3.

### Wettability

The wettability of the films was determined by measuring contact angle (CA) following a sessile drop test.^41^ All films exhibited comparable wettability, with an average water CA of 82.71 ± 8.42° (Figure 9). PCL films showed a higher standard deviation compared to the rest of the films, 87.18 ± 20.7°, which could be explained by the different use of solvent during the casting process, leading to a different evaporation rate and, therefore, differences in the roughness of the films and CA. CA values below 90° indicate a slightly hydrophilic surface of the films^42^ due to the presence of LN. PCL films have been reported to have a CA of 85.08 ± 0.4° when cast using chloroform as a solvent; and 105.83 ± 0.36 with tetrahydrofuran.^43^ PLGA sheets have been reported with a CA of 77.31°, but with the possibility of higher CA (91.58, 97.82, and 99.42°) when imprinted with nanopatterns of 0.5, 1, and 2 μm, respectively.^44^ Superhydrophobic PLA films (CA 153°) have been manufactured using 3D-printed nanopatterns.^45^

**Figure 9:**
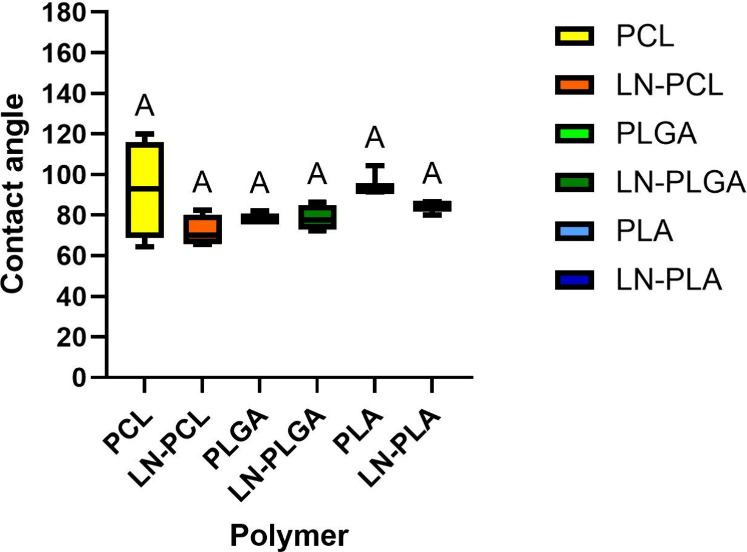
Contact angle analysis of polymer films. Tukey test differences were indicated by *P* < 0.05, *n* = 3.

A comparison with the literature indicates the potential to form films of a lower and tunable CA by grafting LN to polyesters. Other methods of controlling the CA include changing the solvent used in the forming process and modifying the topology of the surface of the films, which was also demonstrated in our previously published work on films with different roughness coefficients.^33,40^

### Water Vapor Permeability

Barrier properties are crucial for any packaging application, and it is one of the most important characteristics for biodegradable materials because water plays a role in food spoilage reactions, loss of physical properties, and dehydration, and these can be affected themselves.^2^ WVP determines the amount of moisture transfer between the environment and the product.^46^ Usually, a lower WVP is required as a higher humidity inside the package can lead to damage, loss of quality, and degradation. Fruits and vegetables are particularly susceptible to water loss, leading to postharvest deterioration.^47^

WVP was measured following the standard cup method.^26^ All films showed a lower weight gain over the 10-day period compared to the control cups, which increased on average by 3.8 ± 0.5 g at the end of the experiment. In comparison, polymer films exhibited a weight gain over time as follows, PCL (1.0 ± 0.4 g), LN-PCL (0.9 ± 0.4 g), PLGA (0.5 ± 0.03 g), LN-PLGA (0.8 ± 0.4 g), PLA (1.0 ± 0.5 g), and LN-PLA (1.6 ± 0.2 g) (Figure 10).

**Figure 10:**
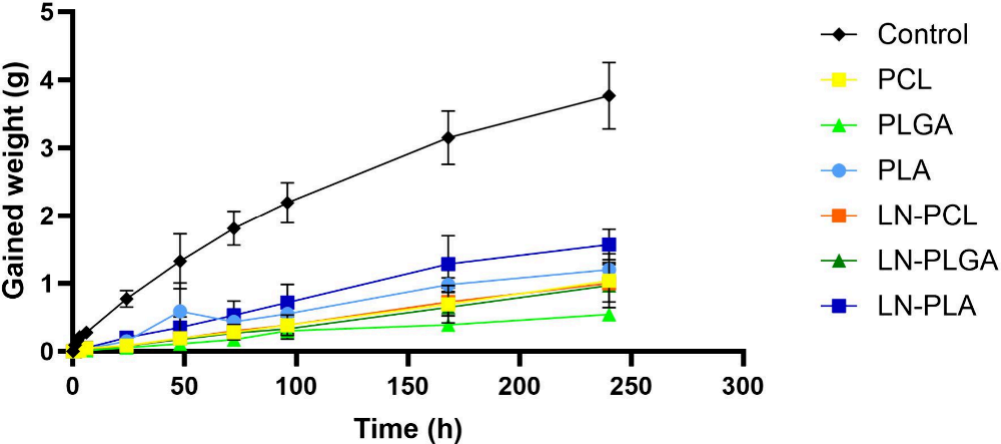
Weight gain over time for LN-(PCL/PLGA/PLA) polymers, *n* = 3.

Neat polymer films showed on average lower permeance compared with their lignin-grafted counterparts. Among the polymers, only PCL and LN-PCL did not show a significant difference in permeance, PCL measuring 0.89 × 10^–7^ g/Pa*s*m^2^ and LN-PCL 0.95 × 10^–7^ g/Pa*s*m^2^. Lignin showed the highest effect in changing the permeance of PLGA films with PLGA films having the lowest permeance of 0.49 × 10^–7^ g/Pa*s*m^2^ and LN-PLGA of 1.0 × 10^–7^ g/Pa*s*m^2^, most likely due to the difference in their thicknesses. Likewise, PLA measured 1.2 × 10^–7^ g/Pa*s*m^2^, significantly lower than LN-PLA films, which had a permeance of 2.0 × 10^–7^ g/Pa*s*m^2^ (Figure 11).

**Figure 11:**
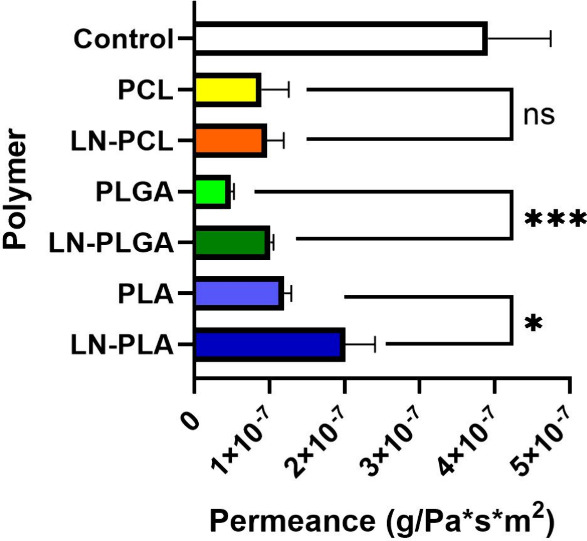
Permeance of LN-(PCL/PLGA/PLA) polymers. Multiple *t*-test analysis results: “ns” = *P* < 0.05, “*” = *P* > 0.05, “**” = *P* > 0.005, and “***” *P* > 0.0005; *n* = 3.

Overall permeability was similar across polymers, and PCL (7 × 10^12^ ± 3.8 × 10^–12^g/Pa*s*m) was not significantly different from LN-PCL (8.5 × 10^–12^ ± 2.4 × 10^–12^g/Pa*s*m). Similarly, PLGA (5 × 10^12^ ± 3.8 × 10^–13^ g/Pa*s*m) and LN-PLGA (3.6 × 10^12^ ± 3.9 × 10^–13^ g/Pa*s*m) showed nonsignificantly different permeability among the films. Lignin impacted only permeability of PLA and LN-PLGA films, significantly. Permeability of PLA was 6.7 × 10^12^ ± 1.8 × 10^–13^g/Pa*s*m, while that of LN-PLA films was 1.5 × 10^11^ ± 1.9 × 10^–12^g/Pa*s*m (Figure 12). LN-PLA film’s increase in permeability is likely caused by the loss of crystallinity in the polymer, which causes an increase in the mobility of the polymer chains helping to the diffusion of water in the film.^48^

**Figure 12:**
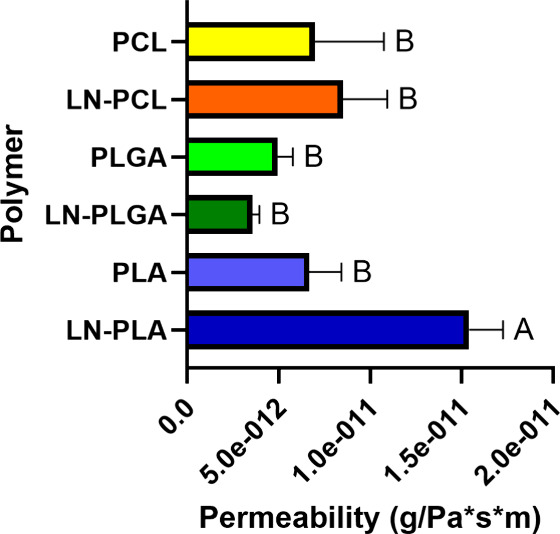
Permeability of LN-(PCL/PLGA/PLA) polymers. Tukey test differences were indicated by *P* < 0.05, *n* = 3.

LN-PCL/PLGA/PLA has comparable permeability to other packaging materials. For example, chitosan films were prepared in aqueous solutions containing 2% (v/v) of either malic or lactic acid. These films showed a permeability of 3.87 × 10^–11^ ± 1.14 × 10^–12^ g/Pa*s*m for films with malic acid and 5.10 × 10^–11^ ± 5.91 × 10^–12^ g/Pa*s*m for films with lactic acid.^2^ Other packaging polymers like PHBV (polyhydroxyalkanoates copolymer) with 8 mol % valerate) and PET (polyethylene terephthalate) show similar permeability (3.0 × 10^–12^ and 5.5 × 10^–12^ g/Pa*s*m) respectively.^49^

## Conclusions

Although numerous studies have investigated the incorporation of lignin into polymer matrices, the covalent grafting of lignin onto polymers prior to film formation remains relatively unexplored, and it is proposed in this study. In summary, lignin was successfully grafted with PCL, PLGA, and PLA and formed into films of different mechanical, chemical, and barrier properties. Thermal analysis showed that glass transition temperature was not affected by the lignin. Tensile strain at break point decreased while yield strength and Young’s modulus increased for LN-PCL and LN-PLGA. The LN-PLA polymer exhibited no significant ductility changes and a decrease in yield strength and Young’s modulus compared to neat PLA. All LN-PCL/PLGA/PLA films had a significantly higher UV absorption compared to their unaltered counterparts. Lignin showed no effect on the wettability of the polymers based on the contact angle results. Permeability of the films was not affected by lignin grafting with the exception of LN-PLA films, which showed an increase in the permeability of the films. Overall, lignin helped increase the UV-shielding properties of the films. LN-grafted polymer films showed similar attributes to neat polymers. It can be said that lignin can be used in packaging materials to increase UV-barrier properties without significantly compromising their other characteristics. Packaging applications typically demand materials with higher tensile strength, improved ductility, and high glass transition temperatures to withstand handling and transportation conditions, properties not fully met by the films developed herein. Some ways to address these limitations may require incorporating these materials into composite materials, applying further chemical modifications, or utilizing them as coatings rather than films to take advantage of their properties without relying on the mechanical properties. Despite these challenges, biodegradability remains a key advantage of these materials, offering a pathway to reduce plastic waste and decrease reliance on petroleum-based polymers. Furthermore, lignin grafting has been shown to enhance UV-shielding properties, which is beneficial for protecting photosensitive contents and enabling resistance to UV sterilization without significant degradation of the material.
